# Secretion of One Adipokine Nampt/Visfatin Suppresses the Inflammatory Stress-Induced NF-*κ*B Activity and Affects Nampt-Dependent Cell Viability in Huh-7 Cells

**DOI:** 10.1155/2015/392471

**Published:** 2015-02-26

**Authors:** Yi-Ching Lin, Hui-Chung Wu, Chen-Chung Liao, Yi-Chih Chou, Shwu-Fen Pan, Chi-Ming Chiu

**Affiliations:** ^1^Department of Biotechnology, Ming Chuan University, Guishan 333, Taiwan; ^2^Proteomics Research Center, National Yang-Ming University, Taipei 11221, Taiwan

## Abstract

Nampt/visfatin acts in both intracellular and extracellular compartments to regulate multiple biological roles, including NAD metabolism, cancer, inflammation, and senescence. However, its function in chronic inflammation and carcinogenesis in hepatocellular carcinoma (HCC) has not been well-defined. Here we use Huh-7 hepatoma cells as a model to determine how Nampt/visfatin affects cellular survival under oxidative stress. We found that the transition of Nampt/visfatin from intracellular into extracellular form was induced by H_2_O_2_ treatment in 293T cells and confirmed that this phenomenon was not due to cell death but through the secretion of Nampt/visfatin. In addition, Nampt/visfatin suppressed cell viability in oxidative treatment in Huh-7 cells and acted on the inhibition of hepatoma cell growth. Oxidative stress also reduced the Nampt-mediated activation of NF-*κ*B gene expression. In this study, we identify a novel feature of Nampt/visfatin which functions as an adipokine that can be secreted upon cellular stress. Our results provide an example to understand how adipokine interacts with chemotherapeutic treatment by oxidative stress in HCC.

## 1. Introduction

Nicotinamide adenine dinucleotide (NAD) is critical for energy homeostasis and mediates a variety of biological activities including inflammation, circadian rhythm, extension lifespan, and cancer [1–5]. Depletion of NAD in myeloma cancer cells increased cell death via autophagy [6, 7]. The salvage pathway for NAD synthesis is regarded as a major way to avoid the depletion of physiological NAD level in mammals [8]. One mammalian enzyme, nicotinamide phosphoribosyltransferase (Nampt), is a rate-limiting enzyme for NAD biosynthesis. It has been demonstrated that Nampt activity is mainly present in adipose tissue, macrophage, hepatocytes, and cancer cells [9]. It is noteworthy that Nampt gene expression appears to be associated with carcinogenic and inflammatory diseases [10, 11]. Inhibition of Nampt by its specific inhibitor FK866 showed an anti-inflammatory activity [12]. Thus, understanding the linkage between NAD biosynthesis and inflammatory stress may resolve the problems associated with the chronic inflammation induced cancer formation.

Besides acting as one NAD synthetic enzyme, Nampt is also called visfatin due to the role of its adipokine activity [13]. A variety of clinical evidence indicates Nampt/visfatin is an important adipokine involved in metabolic disorders [4, 5]. Thus, Nampt/visfatin contains at least two forms of polypeptides. One is an intracellular enzyme called iNampt. The other one is an extracellular factor named eNampt. Several lines of evidence provided the connection between iNampt and NAD-dependent deacetylase/ADP-ribosylase (Sirt1). iNampt has been recognized to mediate Sirt1 to regulate the replication life-span [3]. Reduction of iNampt expression in smooth muscle cells (SMCs) impairs cell survival, whereas overexpression of iNampt upregulates cellular NAD level, induces Sirt1 activity, and then promotes cell maturation [14]. iNampt also protects cardiac myocyte cells from death induced by PARP through NAD dependent activation of Sirt1 [15]. iNampt level can be augmented by genotoxic stress to prevent the depletion of mitochondrial NAD level and then assisted cell survival via Sirt3/4 dependent activation [16, 17].

Despite iNampt being confirmed as one enzyme of NAD salvage pathway to protect cells from apoptosis, the role of eNampt remains controversial. Several studies indicate that eNampt is released from differentiated adipocytes as well as hepatocytes through a non-classical secretory pathway [18–20] and Nampt can exert insulin-mimetic effects* in vitro* or* in vivo* [21]. However, some studies proposed that eNampt-mediated robust NAD biosynthesis might be critical for pancreatic *β* cell in glucose homeostasis [18] rather than the direct insulin-like action [22]. In macrophages, eNampt promotes cell survival to ER stress induced by obesity associated disorders through the activation of IL-6/STAT3 autocrine pathway [23].

Nampt has such a variety of biological roles and much attention appears to focus on the effect that how Nampt prevent an organism from damage of different stress generated via metabolic disorders, aging, and stress from genotoxic drugs for inflammation and cancer therapy. Thus, reports have indicated several anti-Nampt activity compounds and they can act as anti-cancer drugs. For example, APO866 (FK866) as well as CHS-828 has potent antitumor effect against hematologic malignancies [24, 25]. Two other potent Nampt inhibitors, GMX1778 and CB-30865, may have potential for therapeutic candidates to treat certain cancers [26, 27]. The relation of Nampt and cancer has also been mentioned that prostate cancer has higher level of Nampt expression and may enhance cell survival and stress response [28]. However, less study has investigated the role of Nampt in HCC (hepatocellular carcinoma). Thus, we tried to understand the response of cellular Nampt under oxidative stress and the possible role of Nampt in the inflammation state of liver cancer cells.

## 2. Materials and Methods

### 2.1. Cell Culture and Transfection

Human kidney 293T and hepatoma Huh-7 cell lines were grown in DMEM medium with 10% fetal bovine serum at 37°C, 5% CO_2_ incubation. 293T cells were transiently transfected with FLAG-tag Nampt/pCMV2B (a gift from the laboratory of Dr. SC Lee, NTU, Taipei) or pEGFP-N1 (Clontech Laboratories, Takara Bio, Inc., Japan) by calcium phosphate mediated transfection method. Huh-7 cells were transiently transfected with the same plasmids using PolyJet* in vitro* DNA transfection reagent (SignaGen, MD, USA). The siRNAs for Nampt were purchased from Santa Cruz biotech and then transfected into Huh-7 cells via GenMute siRNA transfection reagent (SignaGen, MD, USA).

### 2.2. Cell Treatment

293T cells were treated with H_2_O_2_ at different dosages while Huh-7 cells were treated with 200 *μ*M H_2_O_2_ for 6 hours. In reporter assay experiment, Huh-7 cells were treated with 10 nM FK866 (Sigma-Aldrich, MO, USA) 2 hours before the oxidative treatment.

### 2.3. Protein Lysate Preparation, Immunoprecipitation, and Immunoblot Analysis

Cells were lysed in a buffer containing 10 mM Tris pH 8.0, 20 mM KCl, 0.5 mM EDTA, 200 mM NaCl, 0.1% Triton ×100. Identical amount of cell lysates proceeded SDS-PAGE and then immunoblot analysis by anti-Nampt/PBEF antibody (C20), anti-GADPH (1D4), anti-*β*-actin (C4) (Santa Cruz Biotech, CA, USA), and anti-caspase 9 (N2C3) (GeneTex, CA, USA) antibodies. The antibody for caspase 9 only detects the intact (inactive) form of enzyme. The cell culture medium from two 10 cm cell culture dishes was used for immunoprecipitation by anti-FLAG (M2) agarose resin (Sigma-Aldrich, MO, USA) and then conducted immunoblot analysis.

### 2.4. Cell Viability Assay

Transfected cells were treated with H_2_O_2_ for 6 hours and then replaced with normal culture medium for the following incubation. After 1, 2, 4, and 5 days of culture, cell proliferation activity was determined by a tetrazolium compound MTS purchased from Promega (CellTiter 96 AQueous One Solution Reagent) following the procedures described in technical bulletin.

### 2.5. Protein Identification and Quantitation by Mass Spectrometric Analysis

Proteins from anti-FLAG antibody immunoprecipitates of 293T transfected lysates were resolved in SDS-PAGE. Gel was stained with Coomassie brilliant blue. Each gel lane was sliced into 10 fractions based on molecular weight. The gel slices were destained in a solution of 25 mM NH_4_HCO_3_ and 50% (v/v) acetonitrile (1 : 1). The dried slices were reduced by 2% *β*-mercaptoethanol and then alkylated with 10% (v/v) vinylpyridine in 25 mM ammonium bicarbonate/50% acetonitrile for 20 min at room temperature. The slices were digested by 100 ng of modified trypsin (Promega, Mannheim, Germany) in 25 mM NH_4_HCO_3_ at 37°C overnight. The tryptic peptides were solubilized in 0.1% formic acid and then injected into a nanoflow HPLC system (Agilent Technologies 1200 series, Waldbronn, Germany) coupled to an LTQ-Orbitrap Discovery hybrid mass spectrometer (Thermo Electron, Waltham, Massachusetts, USA). The RAW data were converted by the Xcalibur 2.0.7 SR1 package and an in-house program into DTA files. All DTA files were analyzed by TurboSequest software to match the peptides from human protein sequence database. The protein was identified at least two peptides matched and then the peptide' Xcorr scores was higher than 2.5. Mass spectral counts were normalized using the sum of the spectral counts from each biological sample for quantitative analyses. The relative protein amount is quantified by normalized spectral counts [29].

### 2.6. Luciferase Reporter Assay

Huh-7 cells were cotransfected with NF-*κ*B derived luciferase (pGL4.32[*luc*2P/NF-*κ*B-RE/Hygro]) and CMV-Renilla (pGL4.75[*hRluc*/CMV]) vectors (Promega, Mannheim, Germany). After oxidative treatment, the cells were incubated about 16 hours for reporter gene expression. The cells were lysed by Dual-Luciferase Reporter Assay Kit (Promega, Mannheim, Germany). The relative luciferase activity of 10 *μ*g lysate was detected by FB12-single tube luminometer (Berthold Detection System, Pforzheim, Germany).

### 2.7. Statistical Analysis

Statistical data were calculated by Microsoft Excel 2010 software. Data are presented as the mean ± standard error of the mean (SEM). The significance was showed using Student's *t*-test. § indicates *P* < 0.1; ∗∗ indicates *P* < 0.05; ∗∗∗ or ### indicates *P* < 0.01 compared to the respective control as indicated in legend. Each experimental data consists of three individual replicates.

## 3. Results

### 3.1. Oxidative Stress Leads to the Release of Nampt/Visfatin from Cells

**Figure 1:**
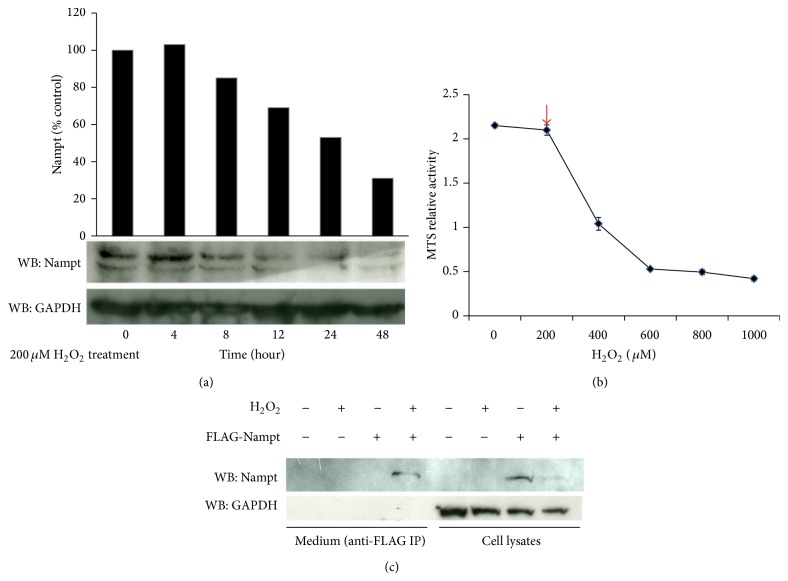
Nampt/visfatin releases from cells after H_2_O_2_ treatment. (a) Huh-7 cells were treated with 200 *μ*M H_2_O_2_ for 6 hours and then its medium was replaced with normal culture medium for additional culture of 4, 8, 12, 24, and 48 hours. The cells were harvested and identical amount of their total lysates were collected for Western blot analysis using anti-Nampt and anti-GAPDH antibodies, respectively. The relative intensity of Nampat/GAPDH ratio is converted into percentage for quantitative analysis. The similar procedures were performed at least three times to verify their reproducibility. (b) Huh-7 cells were treated with different dosages of H_2_O_2_ for 6 hours and then changed medium for additional 24-hour incubation. Cell viability was performed by MTS assay. The standard deviation was shown by three independent experiments. Red arrowhead represented as the H_2_O_2_ concentration used for the following experiments for low dosage of oxidative stress. (c) FLAG-Nampt plasmid was transiently transfected into 293T cells. Next, transfected cells were treated with 200 *μ*M H_2_O_2_ for 6 hours and then change medium for additional one-day culture at 37°C. The cell lysates were isolated for Western blot analysis while the culture medium was collected for immune-precipitation using anti-FLAG immune-affinity resin. Anti-GAPDH antibody was served as lysate loading control. Similar results have reproduced at least five times to make sure of the reproducibility.

Liver has been demonstrated as major source of highly expressed Nampt and the role of Nampt/visfatin in hepatoma cells is less characterized, we try to determine whether the cellular level of Nampt is affected by oxidative stress. This stress may reflect the physiological inflammatory state of liver during carcinogenesis. Huh-7 cell line was initially used as one model system to explore the response. Our observation indicated that the cellular Nampt (iNampt) level was decreased following the treatment of low concentration of H_2_O_2_ in Huh-7 hepatoma cells for 48 hours (Figure 1(a)). To verify the specificity of Nampt secretion, we examined the cell viability using MTS assay under different dosages of H_2_O_2_ treatment to determine the cell damage state. We observed after 24-hour treatment the cell viability at low dosage of H_2_O_2_ (200 *μ*M) in Huh-7 cells has been less affected compared with higher dosage of H_2_O_2_ treatment (400 *μ*M) (Figure 1(b)). The result suggested that the 200 *μ*M of H_2_O_2_ will not damage the intact cell state probably on their membrane structure leading to the leakage of cellular proteins. Propidium iodide (PI) staining of nonfixed Huh-7 cells provided additional evidence that lose dosage of H_2_O_2_ treatment can prevent PI nuclear staining dye from penetrating into nonfixed cells after additional 24-hour culture (see Figure S1 in the Supplementary Material available online at http://dx.doi.org/10.1155/2015/392471). Thus, we proposed that the reduction of iNampt is due to the secretion of Nampt. However, the amount of Nampt was not sufficient to be detectable in culture medium of Huh-7 cells; thus we conducted the exogenously expression of FLAG-tag Nampt in 293T cells and then examined the FLAG-iNampt and FLAG extracellular form of Nampt (eNampt) protein using Western blot and immunoprecipitation-immunoblot analysis, respectively. We also found that the level of FLAG-iNampt was reduced under the treatment of H_2_O_2_ in 293T cells. As we expected, the loss of FLAG-iNampt was detected in the culture medium of H_2_O_2_-treated cells (Figure 1(c)).

### 3.2. The Switch of iNampt and eNampt Is Not due to the Cell Death after the Treatment of H_2_O_2_

**Figure 2:**
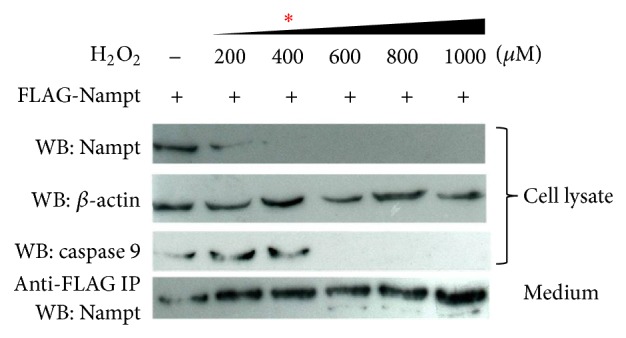
Detection of iNampt and eNampt at different dosages of H_2_O_2_ treatment. The condition of cell treatment and sample preparation were as described in Figure 1. Anti-caspase 9 antibody for catalytic inactive form was applied in immunoblot analysis. The lower panel was shown Western blot detection of Nampt after immunoprecipitation (IP) using anti-FLAG antibody resin from treated condition medium.

To determine the distribution of Nampt between intracellular and extracellular compartment, we increase the concentration of H_2_O_2_ to verify the level of FLAG-eNampt released in culture medium. The level of eNampt was less detectable without H_2_O_2_ addition in 293T cells. It appeared that iNampt prominently decreased upon the treatment of H_2_O_2_ at the concentration of 400 *μ*M. In contrast, eNampt level was increased under the oxidative stress of 200 *μ*M H_2_O_2_ incubation (Figure 2). The level was saturated probably resulting from the limited amount of immunoprecipitated antibody resin. Cleavage of caspase 9 was indicated as the apoptotic effect in 293T cells. Notably, the loss of intact form of caspase 9 in cell lysate of treated cells was observed after we increased the concentration of H_2_O_2_ to 600 *μ*M. It indicated that the concentration of H_2_O_2_ led to the fact that cell death was at least above 600 *μ*M. Thus, the presence of eNampt under the H_2_O_2_ treatment at 200 *μ*M was caused by the secretion of iNampt but not cell death. Similar redistribution of Nampt was also observed in 293T cells treated with etoposide (VP16) (data not shown).

### 3.3. Nampt/Visfatin Potentiates the Suppression of Cell Growth after Oxidative Stress in Hepatoma Cells

**Figure 3:**
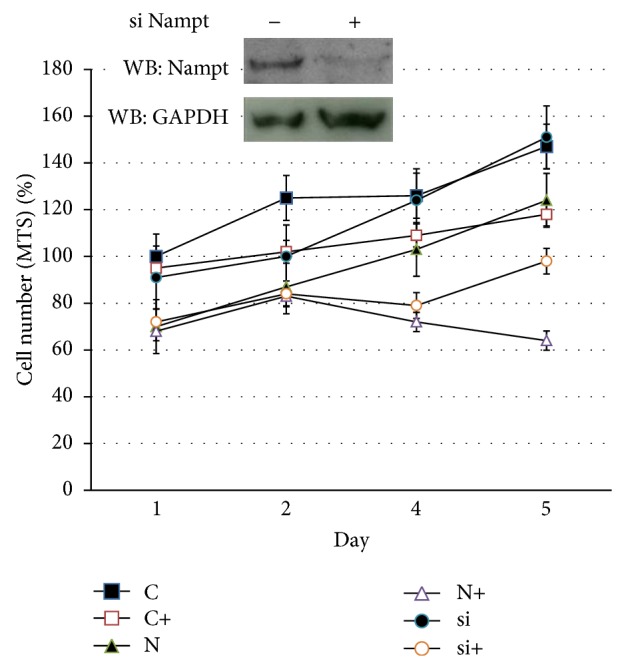
Cell viability assay in Huh-7 cells under oxidative stress. Huh-7 cells were transiently transfected with Nampt plasmid (N) and then siRNAs of Nampt (si) or control (C) were transfected subsequently. After one-day culture, 60–70% confluency of cells was treated with 200 *μ*M H_2_O_2_ for 6 hours as a symbol of “**+**” labeled following the C, N, or si transfected condition. Subsequently, cell culture medium was changed into normal condition for further 1–5 days culture. Cell viability was examined using MTS assay. The experiment was performed in triplicate for SD statistics analysis. The immunoblot result shown in upper left panel indicated the extent of gene suppression of Nampt by siRNA conduction in Huh-7 cells.

Since Nampt secretion resulted from oxidative stress, we would like to understand the biological effects of Nampt under oxidative treatment in hepatoma cells. As we transiently transfected with FLAG-Nampt in Huh-7 cells, less effect has been found in cell viability detected by MTS assay without H_2_O_2_ treatment. Even though the relative activity was somewhat decreased in Nampt transfected cells, the relative curve remained slightly increased after 5-day culture. However, relative cell viability was significantly decreased after 4 days via H_2_O_2_ treatment (Figure 3, triangle). It appeared that the normal cell propagation was lost in Nampt-expressed cells under oxidative stress. Conversely, siRNA for Nampt reversed the suppression of cell normal propagation after H_2_O_2_ treatment (Figure 3, circle). We also determined whether eNampt released from 293T cells after oxidative stress affected the cell viability of Huh-7 cells without oxidative treatment. No prominent cell proliferation activity changed under the conditional medium incubation (data not shown). Recombinant Nampt/visfatin isolated from* E. coli* also obtained similar effect. It suggests that Nampt is required for the inhibition of hepatoma cell growth under oxidative stress.

### 3.4. Nampt/Visfatin Upregulates NF-*κ*B Gene Expression but Suppresses the Effect under Oxidative Stress

**Figure 4:**
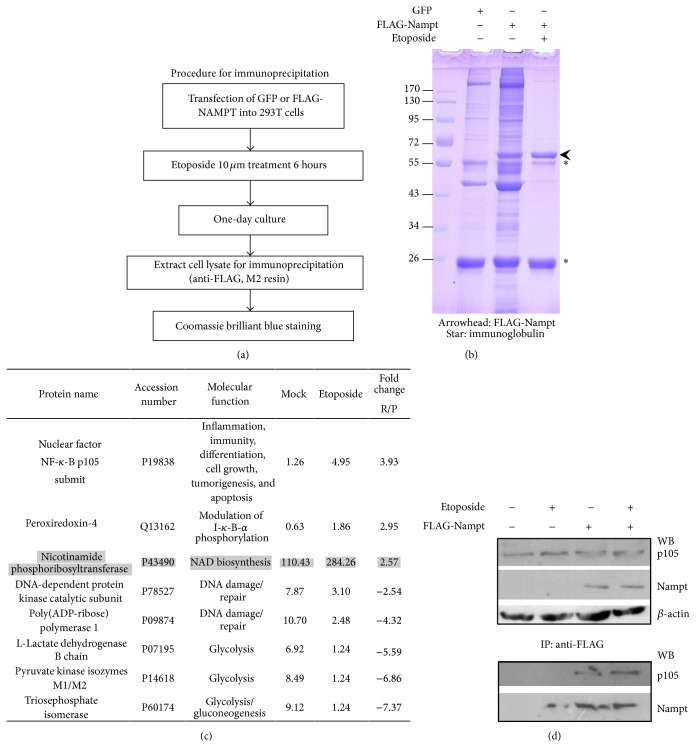
Immuno-protein complex analysis of FLAG-Nampt in 293T cells performed by Orbitrap mass spectrometer. (a) The purification scheme was shown. Transfected 293T cells were treated with genotoxic stress, etoposide 10 *μ*M for 6-hour incubation. GFP transfection was used as negative control. (b) The immunoprecipitates were resolved by SDS-PAGE and then stained with Coomassie brilliant blue. Arrowhead indicates FLAG-Nampt protein position. Star symbol represents as immunoglobulin polypeptides from anti-FLAG affinity gel. (c) The results were shown as protein name, accession number, protein function, the relative values of normalized spectral counts from mock or etoposide-treated sample, and the relative ratio of etoposide untreated versus treated ones quantified using normalized spectral counts. (d) The immunoprecipitation (IP)/Western blot analysis (WB) was performed from the lysates of FLAG-Nampt transfected 293T cells. Upper panel was shown as the one-tenth of direct lysate loading for immunoprecipitation with anti-FLAG affinity gel to verify the protein level of NF-*κ*B p105, FLAG-Nampt, and *β*-actin. Lower panel was represented as the immunocomplex for Western blot analysis of p105 and Nampt proteins.

To investigate the potential candidates affecting cellular Nampt function under oxidative stress, we utilized biochemical approach on the protein complex associated with Nampt/visfatin in 293T cells. Several proteins identified by Orbitrap mass spectrometric analysis present in the immunoprecipitated with FLAG-Nampt. The DNA repair proteins such as DNA-dependent protein kinase catalytic subunit and poly(ADP-ribose) polymerase 1 were decreased in the association with FLAG-Nampt protein complex under genotoxic stress treatment while NF-*κ*B associated proteins p105 and peroxiredoxin-4 enhanced its interaction with Nampt protein complex (Figure 4(c)). Notably, one inflammatory responsive protein NF-*κ*B p105 prominently increased its association with Nampt compared with one in no-etoposide-treated 293T cells. To confirm the above observation, we performed immunoprecipitation of FLAG-Nampt transfected 293T cells. As we immunoprecipitated FLAG-Nampt from 293T cell lysates, p105 protein could be specifically coimmunoprecipitated with Nampt complex. The binding affinity appeared to be slightly enhanced after etoposide treatment (Figure 4(d)). The result was consistent with the mass spectrometric analysis of FLAG-Nampt immunocomplex that p105 of NF-*κ*B protein favored to associate with Nampt complex under oxidative stress.

**Figure 5:**
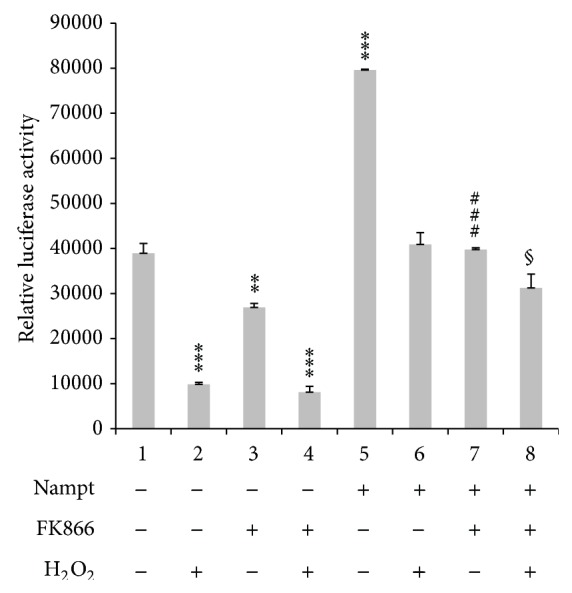
Nampt functional activity on NF-*κ*B gene expression. FLAG-Nampt plasmid as well as NF-*κ*B luciferase reporter vector was transiently transfected into Huh-7 cells. After one-day incubation, the cells were treated with 10 nM FK866 for 1 hour before H_2_O_2_ treatment for 6 hours. Cells were changed into normal medium for 16–18 hours incubation. The lysates were harvested and measured their luciferase activity normalized with CMV-drive Renilla luciferase activity in each transfection. The experiment was performed in triplicate. § *P* value < 0.1 (6 versus 8); ^**^ *P* value < 0.05 (1 versus 3); ^***^ *P* value < 0.01 (1 versus 2, 1 versus 4, 1 versus 5); ^###^ *P* value < 0.01 (5 versus 7).

Next, we conducted NF-*κ*B-drive luciferase reporter assay to examine whether Nampt affected its gene expression. We found that oxidative treatment downregulated NF-*κ*B gene expression in Huh-7 cells. However, NF-*κ*B activity was induced in the presence of Nampt overexpression. As the cells treated with 200 *μ*M of H_2_O_2_, the increase of NF-*κ*B gene expression will be suppressed. Inhibition of Nampt activity by one specific inhibitor FK866 also reversed the induction effect (Figure 5). As we determined the NF-*κ*B reporter activity using higher dosage of H_2_O_2_ (500 *μ*M), low reporter activity is detectable in Huh-7 cells. However, if we examined Nampt-mediated NF-*κ*B gene expression under genotoxic stress using etoposide treatment, we would find that NF-*κ*B gene expression was induced by genotoxic stress (Supplementary Material—Figure S2). The upregulation is also observed in the presence of Nampt overexpression, but the induction cannot be further enhanced after genotoxic stress. The decrease extent of NF-*κ*B activation by FK866 was only affected on the level of Nampt-mediated activation. These results indicated that Nampt specifically mediated NF-*κ*B gene induction. The effect will be downregulated upon oxidative/genotoxic stress consistent with the loss of iNampt under the same treatment. It suggests that Nampt is participated in NF-*κ*B gene expression and affected under oxidative treatment.

## 4. Discussion

Adipokines such as adiponectin as well as leptin have been demonstrated in the obesity-associated disorders, nonalcohol fatty liver disease (NAFLD) and hepatocellular carcinoma (HCC) [30]. Recent study has demonstrated that adiponectin rather than leptin or visfatin (eNampt) is associated with HBV or metabolic induced HCC [31]. However, the biological role of cellular Nampt/visfatin (iNampt) on hepatoma cells has remained to be determined. Here, we explored the biological role of Nampt/visfatin in hepatoma cells and examine the effect under oxidative stress similar to the condition of chronic low-level inflammation. We found that the release of Nampt into medium in the FLAG-tag Nampt overexpressed cell system can be stimulated under H_2_O_2_ treatment. In addition, Nampt is required for Nampt-mediated inhibition of cell growth under oxidative condition. Although Nampt expression activated NF-*κ*B gene expression, inactivation of Nampt by FK866 reduced the effect. Oxidative stress also led to similar effect. Therefore, low level of iNampt caused by oxidative condition may serve as one mechanism in the regulation of inflammatory state and cell viability in hepatoma cells.

Nampt/visfatin has been regarded as one adipokine in the verification of metabolic disease, cancer progression, and chronic inflammation. The role of Nampt/visfatin in hepatocytes has been less addressed and remains to be further determined. Nampt/visfatin secretion has been identified as one nonclassical pathway in adipocytes and hepatocytes [18, 20] whereas no stress response was identified to be involved in the regulation of secretion. Our finding about the release of exogenous expressed Nampt was one unique effect and could be suitable for the detection of stress state* in vivo*. In addition, there is not only oxidative stress but also genotoxic stress that identified the effect. It suggests that the oxidative response seems to be general for any ROS-related stress in the determination of the free radical extent* in vivo*.

Although the level of circulated Nampt (eNampt) under chronic inflammation could be monitored in obesity, diabetes, even metabolic disorders [32, 33], less characterization in biological effect of Nampt/visfatin has reported after the reduction of intracellular Nampt (iNampt) level under stress condition. We identified the decrease of cell viability or cell growth arrest in Nampt expressed cells under oxidative stress. It may imply that either increase of eNampt or decrease of iNampt triggers certain factors in the regulation of cell cycle or mitochondrial enzymes determined by MTS assay. According to the study of visfatin in cell cycle regulation, branched chain amino acids induced p21-mediated cell cycle arrest and then visfatin caused apoptosis in HCC [34]. Nevertheless, further studies are required for the understanding how cellular activities are affected by the Nampt-dependent cell viability in hepatoma cells.

The biochemical interaction of Nampt has been known to form homodimer and no prominent studies have shown its associated proteins. In this study, NF-*κ*B p105 was identified from tag-bait immunoprecipitation of Nampt in 293T cells. Previous research has demonstrated that visfatin induced-ROS generation led to the phosphorylation and activation of NF-*κ*B in C2C12 cells [35]. Our results are consistent with the effect that Nampt can positively affect NF-*κ*B derived reporter whereas oxidative treatment causes the opposite effect. Reasonable possibility is that depletion of iNampt may diminish the positive effect of NF-*κ*B activation. The investigation of cytoplasmic and nuclear distribution of NF-*κ*B regulated by Nampt remained to be elucidated.

Nampt inhibitors such as FK866/APO866 and CHS828 are applied in chemotherapeutic clinical trial in cancer therapy [24, 25]. Other genotoxic drugs that often induce oxidative stress* in vivo* have generally served as chemotherapeutic weapon in clinical therapy. Here we initially observed the effect of cellular response by these two types of drugs applied in the same time. Nampt/visfatin involved in the chronic inflammatory effect may provide us to evaluate the dosage of drugs in the therapeutic procedure. It also gives an example of how an adipokine participates in the chemotherapy to regulate hepatoma cell viability.

Taken together, we utilized an exogenously overexpressed Nampt to examine the release effect of Nampt/visfatin mediated by oxidative stress. The response is different from the protective role of Nampt to promote the cancer cell proliferation [36]. This could explain that the induction of Nampt gene expression is usually observed in oxidative/genotoxic stress in proliferative cancer cells. It could make tumor cells sensitive to the stress microenvironment in the decision of cell arrest or death. Increase of the eNampt/visfatin level might serve as one mechanism to maintain the proliferation of cells under chronic stress condition. Regulation of oxidative NF-*κ*B gene expression might be regarded as the feedback control of oxidative stress. However, the mechanism of how NF-*κ*B is involved in the switch of iNampt/eNampt needs to be determined.

## 5. Conclusions

We identified the release effect of Nampt/visfatin after oxidative treatment. The effect is not due to cell death under low level H_2_O_2_ administration. However, Nampt reduced hepatoma cell viability at the same condition following further incubation. We also found that Nampt activated NF-*κ*B gene expression but the activity was suppressed after oxidative treatment. These results suggest that Nampt might contribute a positive role in cellular inflammation response but the loss of iNampt or the presence of eNampt/visfatin suppresses the cell viability. The possible mechanism may be through the regulation of NF-*κ*B activity to mediate cell death. The study provides a novel role of Nampt/visfatin in the regulation of cell survival under oxidative stress and gives one diagnostic strategy to determine cell viability via NAD relative regulatory mode* in vivo*.

## Supplementary Material

To characterize the cell death of Huh-7 cells under the H_2_O_2_ treatment, PI staining indicated 200μM of H_2_O_2_ addition cannot lead to cell death after one day culture (Figure S1). Propidium iodide (PI) utilized for cell nuclei staining was bought from Sigma-aldrich, MO, USA. The fluorescent analysis was observed by fluorescent microscope BX51 (Olympus). NF- 𝜅B gene expression is regulated by Nampt or genotoxic drug – etoposide (VP-16) was examined using luciferase reporter assay as described in material and method section (Figure S2). Etoposide (VP-16) was bought from Sigma-aldrich, MO, USA.
