# Supplementary material for: Secretion of One Adipokine Nampt/Visfatin Suppresses the Inflammatory Stress-Induced NF-κB Activity and Affects Nampt-Dependent Cell Viability in Huh-7 Cells
Source: Mediators Inflamm. 2015 Feb 26;2015:392471. doi: 10.1155/2015/392471 (PMC4357042; doi:10.1155/2015/392471)

Supplementary materials

Figure S1 Propidium iodide (PI) staining of non-fixed H_2O_2-treated Huh-7 cells.

Huh-7 cells were treated with different dosages of H_2O_2 from 200~1000μM concentration for 6 hours and then changed normal medium for additional 24 hours culture. The cells were washed with PBS twice and following the direct PI dye staining without any permeabilized procedures for normal PI staining for 30 minutes. The cells were observed in lived condition by fluorescent microscopic analysis (Olympus). The bright red signals were shown as the PI dye incorporation into the nuclei of death cells. Low red background was regarded as lived cells exclusive of staining by PI. The images were magnified to 10x40 folds.

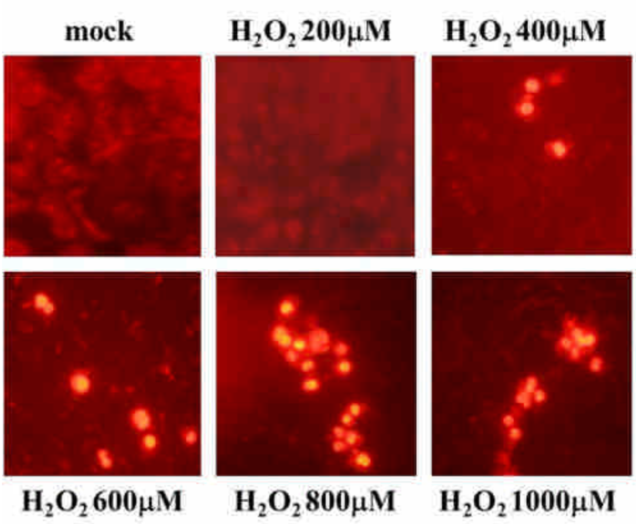

Figure S2 Nampt functional activity on NF-κB gene expression by genotoxic stress.

FLAG-Nampt plasmid as well as NF-κB luciferase reporter plasmid was transfected into Huh-7 cells. Next, the cells were treated with 10nM FK866 and subsequent 10μM etoposide (VP-16) treatment for 6 hours. After 16-18 hours culture, the cell lysates were harvested and measured their luciferase activity normalized with CMV-drive Renilla luciferase activity in each transfection. The experiment was performed in triplicated.
** P value<0.05 (5 vs 7, 6 vs 8); *** P value<0.01 (1 vs 2, 1 vs 5, 1 vs 6)

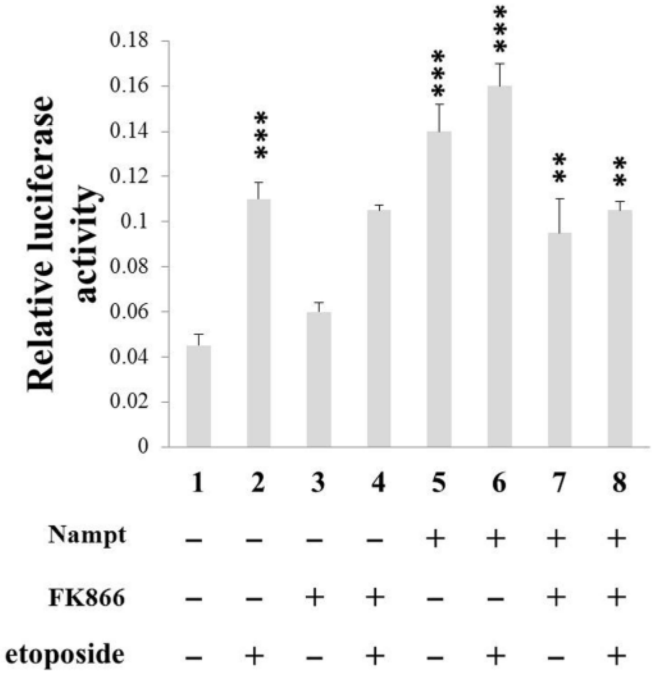

Supplement: Supplementary file 1 — To characterize the cell death of Huh-7 cells under the H2O2 treatment, PI staining indicated 200μM of H2O2 addition cannot lead to cell death after one day culture (Figure S1). Propidium iodide (PI) utilized for cell nuclei staining was bought from Sigma-aldrich, MO, USA. The fluorescent analysis was observed by fluorescent microscope BX51 (Olympus). NF- 𝜅B gene expression is regulated by Nampt or genotoxic drug – etoposide (VP-16) was examined using luciferase reporter assay as described in material and method section (Figure S2). Etoposide (VP-16) was bought from Sigma-aldrich, MO, USA. [file 392471.f1.pdf]
